# Urine L-selectin reflects clinical and histological renal disease activity and treatment response in lupus nephritis across multi-ethnicity

**DOI:** 10.3389/fimmu.2023.1200167

**Published:** 2023-08-31

**Authors:** Yiwei Shen, Kamala Vanarsa, Zhihua Yin, Ting Zhang, Jessica Castillo, Min Dai, Linghua Zou, Ling Qin, Jieying Wang, Qiang Guo, Ramesh Saxena, Michelle Petri, Nan Shen, Zhizhong Ye, Chandra Mohan, Huihua Ding

**Affiliations:** ^1^ Department of Rheumatology, Shanghai Institute of Rheumatology, Renji Hospital, Shanghai Jiao Tong University School of Medicine, Shanghai, China; ^2^ Department of Biomedical Engineering, University of Houston, Houston, TX, United States; ^3^ Department of Rheumatology, Shenzhen Futian Hospital for Rheumatic Diseases, Shenzhen, China; ^4^ Department of Rheumatology, Joint Research Laboratory for Rheumatology of Shenzhen University Health Science Center and Shenzhen Futian Hospital for Rheumatic Diseases, Shenzhen, China; ^5^ Division of Rheumatology, The Second Affiliated Hospital, Zhejiang University School of Medicine, Zhejiang, China; ^6^ Department of Rehabilitation, Shenzhen Futian Hospital for Rheumatic Diseases, Shenzhen, China; ^7^ Department of Nephrology, Shanghai Tenth People’s Hospital, Tongji University School of Medicine, Shanghai, China; ^8^ Clinical Research Unit, Renji Hospital, Shanghai Jiao Tong University School of Medicine, Shanghai, China; ^9^ Division of Nephrology, University of Texas Southwestern Medical Center, Dallas, TX, United States; ^10^ Division of Rheumatology, Johns Hopkins University School of Medicine, Baltimore, MD, United States; ^11^ State Key Laboratory of Oncogenes and Related Genes, Shanghai Cancer Institute, Renji Hospital, Shanghai Jiao Tong University School of Medicine (SJTUSM), Shanghai, China; ^12^ Center for Autoimmune Genomics and Etiology, Cincinnati Children’s Hospital Medical Center, Cincinnati, OH, United States; ^13^ Department of Pediatrics, University of Cincinnati College of Medicine, Cincinnati, OH, United States

**Keywords:** lupus nephritis, L-selectin, urinary biomarker, renal histopathology, treatment response

## Abstract

**Objective:**

There is an urgent need for novel biomarkers in lupus nephritis (LN). We report a non-invasive urinary biomarker, L-selectin, in two independent multi-ethnic cohorts.

**Methods:**

uL-selectin was tested cross-sectionally in a Chinese cohort (n=255) and a US cohort (n=219) of SLE patients and controls using ELISA. A longitudinal cohort includes 20 active Chinese LN patients.

**Results:**

uL-selectin was significantly increased in active LN patients compared to active non-renal SLE, inactive LN, inactive non-renal SLE, chronic kidney disease patients, and healthy controls. uL-selectin positively correlated with global and renal disease activities and was significantly associated with histological activity index and chronicity index (CI). Low uL-selectin was an independent predictor for high CI. During follow-up, uL-selectin levels decreased significantly in the complete renal remission group.

**Conclusion:**

uL-selectin is a novel biomarker of disease activity and renal histopathology in LN across multiple ethnicities. It also reflects treatment response in LN patients during follow up.

## Introduction

Lupus nephritis (LN) is one of the most common and serious manifestations of Systemic lupus erythematosus (SLE), which is more prevalent in African American, Hispanic, and Asian patients. Without prompt diagnosis or proper treatment, ~5~20% of LN patients would proceed to end-stage renal disease (ESRD) within 10 years from the initial diagnosis (1).

Currently, a kidney biopsy is the gold standard for the diagnosis of LN, which guides management strategy. However, various drawbacks limit its application in practice, including its invasive nature with major complications, interobserver variability, and patients’ unwillingness. Although repeat biopsy has been implicated in long term management of LN (2), serial biopsies to monitor renal disease is not always practical. Hence, biomarkers, especially urine biomarkers, have become a promising tool for diagnosing and monitoring disease, evaluating treatment response, and predicting renal flares in LN patients, due to its non-invasive nature and repeatability.

Recent advances in “omics” technologies have changed the strategy of biomarker discovery from a hypothesis-driven approach to an agonistic approach. Previous studies using affinity-based techniques such as antibody-based or aptamer-based assays have identified novel protein biomarkers in urine (3, 4). Whether these biomarkers are robust enough for clinical use is still under investigation. One of the most important investigations is to rigorously validate the screened biomarkers in large-scale studies with multi-ethnic cohorts. Among the recently reported newly discovered urinary biomarkers, L-selectin emerged as a novel urinary biomarker of LN with good potential in distinguishing active LN patients from active non-renal SLE patients in a small validation cohort (4). In the current study, we aim to systematically validate L-selectin as a urinary biomarker of disease activity and treatment response in LN patients across multiple ethnicities, cohorts and test centers, a pre-requisite for eventually using these biomarkers in clinical practice.

## Materials and methods

### Study subjects

#### Cross-sectional cohorts

The cross-sectional study included subjects of two cohorts from three centers. The primary cohort was comprised of 195 Han Chinese SLE patients from the Renji Hospital, Shanghai Jiao Tong University (SJTU) School of Medicine, China, recruited from 2017 to 2019, including 87 biopsy-proven active LN (aLN), 57 active non-renal SLE (aNR), 25 inactive LN (iLN) and 26 inactive non-renal SLE (iNR). All aLN patients in the Chinese cohort had concurrent renal biopsies performed. Additionally, 33 patients with chronic kidney diseases (CKD) and 27 age- and gender-matched healthy subjects were also recruited as disease and healthy controls, respectively. The US-based cohort included 63 SLE patients from the Johns Hopkins University (JHU) School of Medicine, Baltimore, MD, United States; 103 SLE patients and 53 healthy controls from the University of Texas Southwestern (UTSW) Medical Center’s Renal Clinic, Dallas, TX, United States, among which 32 SLE patients with active LN also had concurrent renal biopsies performed. The US-based cohort consisted of 34 Caucasian subjects, 114 African American subjects and 71 Hispanic subjects (**Figure S1**).

SLE patients with clinical components of Systemic Lupus Erythematosus Disease Activity Index-2k (cSLEDAI-2k) ≥ 4 were defined as active SLE, whereas patients with renal SLEDAI (rSLEDAI, refers to the total score of the four kidney-related parameters in SLEDAI) ≥ 4 were classified as aLN patients. Patients with active SLE and no history of renal involvement with rSLEDAI =0 were defined as aNR patients (5). The iLN patients had a history of LN with SLEDAI < 4 and rSLEDAI = 0. The iNR patients had no history of renal involvement with SLEDAI < 4 and rSLEDAI = 0 (**Tables S1-5**). All SLE patients met the 1997 revised American College of Rheumatology (ACR) classification criteria for SLE or 2012 SLICC criteria for SLE (6, 7). Informed consent was obtained from all participants and the study was approved by the ethics committee of Renji Hospital, Shanghai Jiao Tong University School of Medicine, Shanghai, China, and the Institutional Review Boards of the University of Houston, JHU School of Medicine, and UTSW.

#### Longitudinal cohort

Among the 87 active LN patients in the Chinese cohort, 20 patients who were followed up for at least 6 months had urine samples collected and stored at the end of the follow-up, and their clinical and laboratory results were documented and treatment outcomes were also evaluated.

### Disease assessment

Disease activity was assessed by SLEDAI and rSLEDAI. In the SJTU cohort, SLICC renal activity score (SLICC RAS) was also calculated to assess renal activity in aLN patients as previously described (8).

For the longitudinal cohort, treatment response was defined as complete renal remission (CRR), partial renal remission (PRR) or no renal remission (NRR), and the definitions were described in detail in **Supplementary Materials** (9).

### Renal histology

All renal biopsies were documented for LN classes and scored for activity index (AI), chronicity index (CI), and their component attributes by two independent experienced renal pathologists who were blinded to the design of the study, using the 2018 revision of International Society of Nephrology/Renal Pathology Society (ISN/RPS) classification for lupus nephritis (10). For US-based cohort, only AI and CI were recorded without detailed component attributes.

### Assay of urinary L-selectin

Clean-catch midstream urine was collected from each patient in a 50 mL sterile container in the morning. For biopsy-concurrent aLN patients, urine samples were procured within 5 days before kidney biopsy. Urine samples were aliquoted to avoid repeated freeze-thaw cycles and stored at − 80°C. Urinary L-selectin (uL-selectin) was assayed using a commercially available human L-selectin enzyme-linked immunosorbent assay (ELISA) kit (DY728, R&D System; ELH-LSelectin, Raybiotech; 1:20) according to the manufacturer’s instruction. uL-selectin was normalized by urine creatinine using Creatinine Parameter Assay Kit (KGE005, R&D Systems).

### Statistical analysis

Data were analyzed and plotted using SPSS 26, GraphPad Prism 9.0 or R (Version 4.2.0). Data were expressed as mean (SD) for continuous variables with normal distribution, median (interquartile range (IQR)) for continuous variables with non-normal distribution and counts and percentage for dichotomous variables. The Kolmogorov–Smirnov tests established the normality of data. Group comparisons were made using the Mann-Whitney U test, Kruskal–Wallis, Wilcoxon matched-paired signed rank test, Chi-Squared or Fisher exact tests as appropriate. Non-parametric Spearman’s method was performed for correlation analysis. Receiver operating characteristic (ROC) curve and areas under curve (AUC) were performed as appropriate. Correlation heatmap was generated using corrplot and Hmisc packages in R.

Patients of 87 biopsy-proven LN in the Chinese cohort were dichotomized according to median renal histological activity (median of AI, 6) and median renal histological chronicity (median of CI, 3). The association between uL-selectin levels at the baseline and high AI (AI > 6) or high CI (CI > 3) was investigated using univariate and further multivariate logistic regression by controlling the effect of confounding variables, including age, gender, SLE disease duration, LN disease duration, 24h proteinuria, eGFR and SLEDAI. A two-tail P value less than 0.05 was considered significant.

## Results

### uL-selectin was exclusively elevated in active lupus nephritis

**Figure 1:**
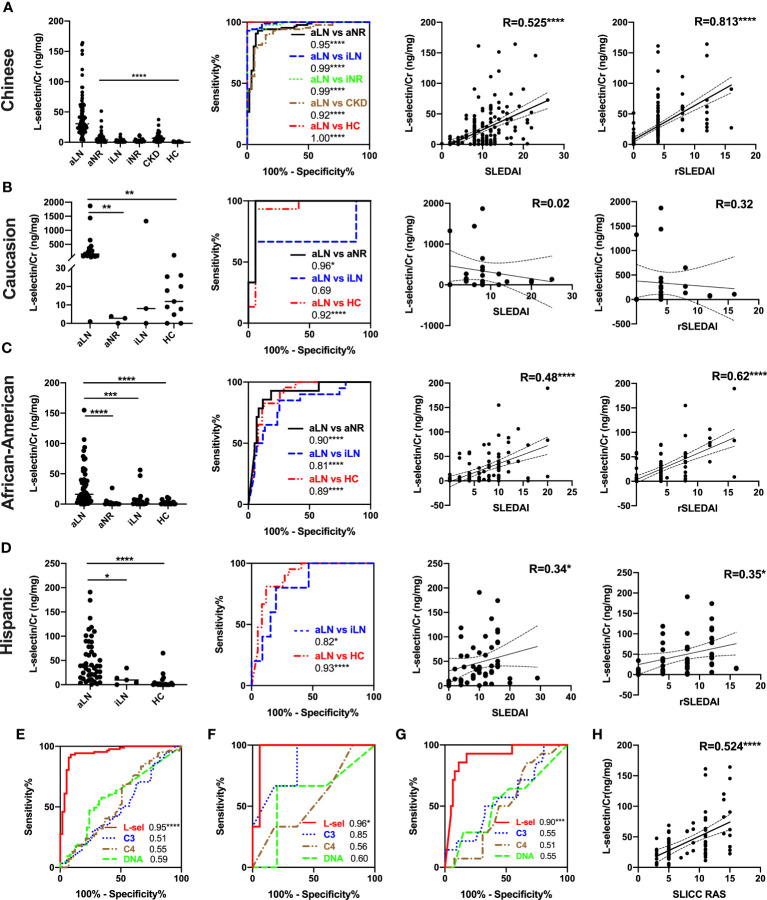
Comparison of uL-selectin levels among subject groups and their correlation with clinical indices among Asian (all were Chinese) **(A, E, H)**, non-Hispanic Caucasian **(B, F)**, non-Hispanic African American **(C, G)** and Hispanic **(D)** subjects. Kruskal-Wallis test and Dunn *post hoc* multiple comparisons test were used among subgroups across ethnicities. ROC curve analyses were performed, demonstrating the ability of uL-selectin to discriminate aLN from other subgroups **(A–D)** and its better performance in discriminating aLN from aNR than conventional biomarkers (C3, C4 and anti-dsDNA antibody) in the Asian **(E)**, Caucasian **(F)** and African American **(G)** groups. Values in the plot indicate areas under curve (AUC). uL-selectin was correlated significantly with SLEDAI, rSLEDAI in the Asian, African American, and Hispanic groups **(A, C, D)**, as well as with SLICC RAS in the Asian group **(A, H)**. With respect to the data from the African American subjects, the shown analyses have been executed after removing 2 outliers. Whereas the mean ± SD of uL-selectin level in the rest of the patients was 29 ± 31.5 (ng/mg), the mean in these 2 outliers was 3533.7 (ng/mg). Inclusion of both subjects yielded correlation coefficients of 0.54 and 0.61 with SLEDAI and rSLEDAI, respectively (not shown). aLN, active lupus nephritis; HC, healthy control; aNR, active non-renal; iLN, inactive lupus nephritis; iNR, inactive non-renal; CKD, chronic kidney disease; L-sel, urinary L-selectin adjusted by creatinine; DNA, anti-dsDNA antibody; R, Spearman’s correlation coefficient; *P<0.05, **P<0.01, ***P<0.001, ****P<0.0001.

In the Chinese cohort, uL-selectin levels were increased exclusively in aLN patients when compared with aNR patients, iLN patients, iNR patients, HC, or CKD patients (all p < 0.0001, **Figure 1A**). uL-selectin could significantly discriminate aLN patients from other groups of patients (all p < 0.0001, **Figure 1A**). uL-selectin outperformed conventional markers including C3, C4 and anti-dsDNA antibody in discriminating aLN patients from aNR patients (**Figure 1E**).

The US-based cohort was comprised of 219 subjects including 121 aLN patients, 17 aNR patients, 28 iLN patients and 53 HC. Results are presented parsed by ethic/racial groups – non-Hispanic Caucasians, non-Hispanic African Americans and Hispanics. In all of three subgroups, uL-selectin levels were significantly elevated in aLN patients when compared with HC (all p < 0.001, **Figures 1B, C**). uL-selectin levels further discriminated aLN from aNR patients in the Caucasian group (p < 0.01, **Figure 1B**) and the African American group (p < 0.0001, **Figure 1C**); aLN patients had higher uL-selectin levels than iLN patients in the African American group (p < 0.001, **Figure 1C**) and the Hispanic group (p < 0.05, **Figure 1D**). uL-selectin showed a better capability than conventional biomarkers in discriminating aLN and aNR patients in the Caucasian and African American groups (**Figures 1F, G**).

### uL-selectin correlated with disease activity and other clinical characteristics

In the Chinese cohort, there is significant correlation between uL-selectin and disease activity indices, including SLEDAI, rSLEDAI and SLICC RAS (all p < 0.0001, **Figures 1A, H**) as well as 24h proteinuria, eGFR, serum Cr, complements, ESR and hemoglobin levels (all p<0.05) (**Table S6**). Furthermore, uL-selectin levels were associated with lymphadenopathy, Raynaud’s phenomenon and serous effusion in SLE patients (**Table S7**).

In the US-based cohort, uL-selectin was positively correlated with SLEDAI and rSLEDAI in the African American (both p < 0.0001) and Hispanic (both p < 0.05) groups. But there was no significant correlation between uL-selectin levels and disease activity in the Caucasian group (**Figures 1B–D**). uL-selectin levels also correlated with 24h proteinuria, anti-dsDNA titers, serum C3, C4 and ESR (**Table S8**).

### uL-selectin reflected concurrent renal pathological indices

**Figure 2:**
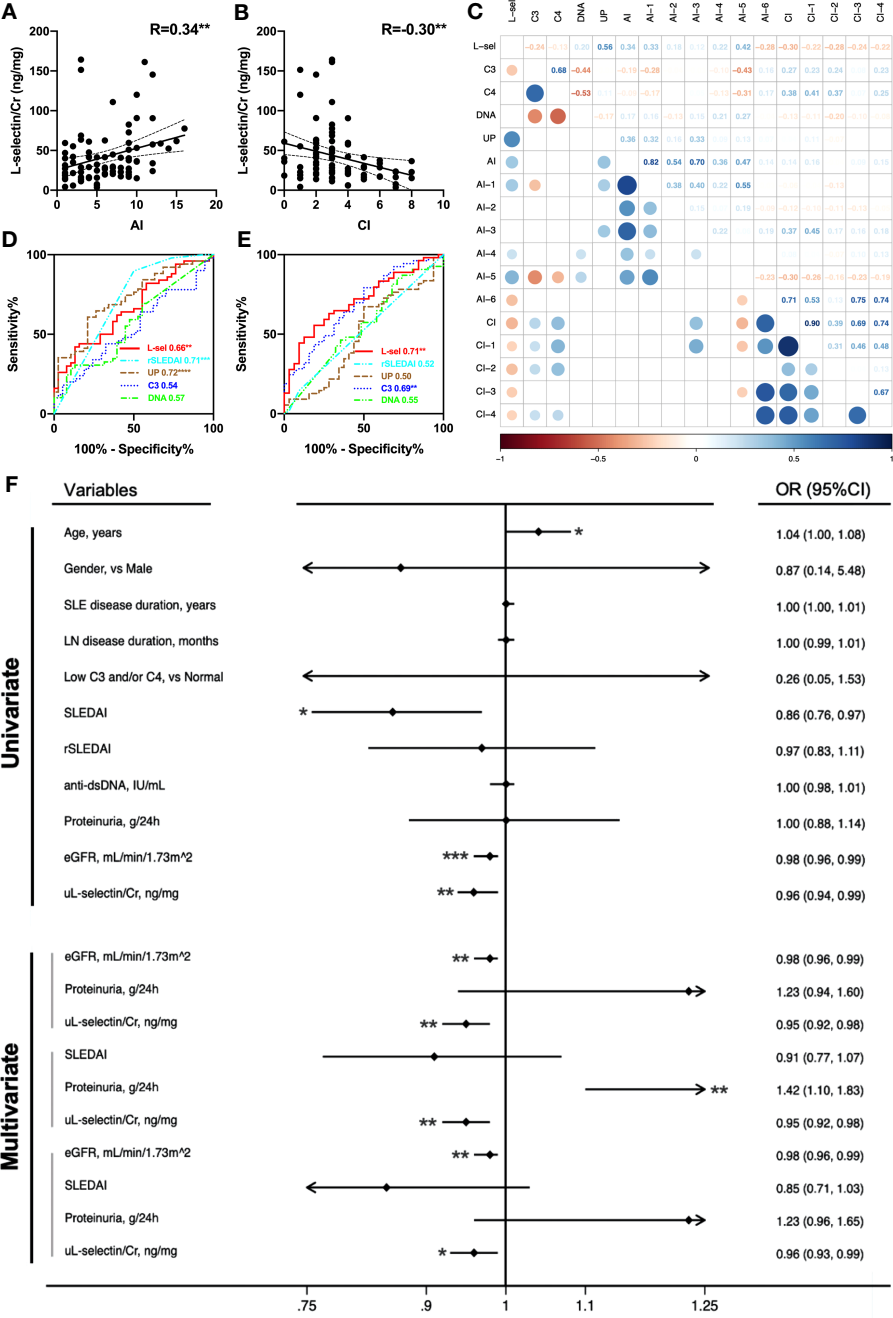
uL-selectin reflects concurrent renal pathology indices in LN. uL-selectin in LN patients correlated with AI **(A)** and CI **(B)** by Spearman correlation analysis. **(C)** Correlation matrix for comparison of uL-selectin and conventional metrics (serum C3, C4, anti-dsDNA and 24h proteinuria) with renal pathology AI, CI and their component attributes. The numbers in squares (upper right), and the colors and size of circles (lower left) all represent the corresponding Spearman’s correlation coefficient. The upper right and lower left halves of the plot depict the same results across an imaginary diagonal. The circles (lower left) were removed where the corresponding P value for the correlation coefficient exceeded 0.05. **(D)** uL-selectin could discriminate high AI (AI>6) from low AI (AI ≤ 6), and **(E)** could also differentiate high CI (CI>3) from low CI (CI ≤ 3), compared with conventional disease indices. Values in the plot indicate areas under curve. **(F)** The forest plot summarizes results from univariate and multivariate logistic regression analysis for high renal pathology CI scores. Lower urinary L-selectin was associated with an increased risk of high CI (CI>3) even after adjusting for age, gender, SLE disease duration and LN disease duration. OR values in multivariate logistic regression were adjusted OR values. AI, activity index; CI, chronicity index; R, Spearman’s correlation coefficient; L-sel/uL-selectin/Cr, urinary L-selectin adjusted by creatinine; DNA, anti-dsDNA antibody; UP, 24-hour urine protein quantity; AI-1, Endocapillary hypercellularity; AI-2, Neutrophils/karyorrhexis; AI-3, Cellular/fibrocellular crescents; AI-4, Fibrinoid necrosis; AI-5, Hyaline deposits; AI-6, Interstitial inflammation; CI-1, Glomerulosclerosis; CI-2, Fibrous crescents; CI-3, Interstitial fibrosis; CI-4, Tubular atrophy; eGFR, estimated glomerular filtration rate; OR, odds ratio; 95% CI, 95% confidence interval. *P<0.05, **P<0.01, ***P<0.001.

In the 87 active LN patients with concurrent renal biopsies in the Chinese cohort, uL-selectin levels were elevated in each LN pathology class when compared with HC (p<0.0001). There was a trend for higher uL-selectin levels in proliferative LN (III ± V & IV ± V) patients than in non-proliferative LN (II & V) patients, but no statistical significance was observed (**Figure S2**). Importantly, uL-selectin positively correlated with AI (r = 0.34, p < 0.01) and negatively correlated with CI (r = -0.30, p < 0.01) in renal histopathology (**Figures 2A, B**). When we looked into the detailed aspects of AI and CI, uL-selectin correlated significantly with endocapillary hypercellularity, fibrinoid necrosis, wire loop deposits and interstitial inflammation of AI and with glomerulosclerosis, fibrous crescents, interstitial fibrosis and tubular atrophy of CI (**Figure 2C**; **Table S9**). ROC analyses confirmed the potential of uL-selectin to discriminate high AI (AI>6) from low AI (AI ≤ 6) (p<0.01, **Figure 2D**), and to differentiate high CI (CI>3) from low CI (CI ≤ 3) (p<0.01, **Figure 2E**). uL-selectin enhibited a similar capability to 24h urine protein and rSLEDAI in discerning high and low levels of AI. Additionally, uL-selectin displayed an exceptional proficiency when distinguishing between high and low levels of CI. Univariate logistic regression analysis was performed to discover potential risk factors for high CI. The results showed that lower uL-selectin (p = 0.005), higher age (p = 0.047), lower eGFR (p < 0.001) and lower SLEDAI (p = 0.014) were associated with high CI (**Figure 2F**; **Table S10**, **Figure S3**). Multivariate logistic regression models constructed for predicting high CI revealed that the addition of uL-selectin to all the evaluated models significantly improved the model fit after adjustment for age, gender, SLE disease duration, LN duration and 24h proteinuria, and its contribution was always significant. (**Figure 2F**; **Table S11**, **Figure S3**).

Correlation analyses of uL-selectin with renal pathology indices were also performed in 32 biopsy-concurrent LN patients from the US-based cohort. As observed in the Chinese cohort, uL-selectin showed significant positive correlation with AI (r=0.47, p<0.01), and negative correlation with CI (not attaining significance) (**Figure S4**). In this cohort, uL-selectin was superior to proteinuria in distinguishing patients with high AI and those with high CI from the controls (**Figure S4**). To evaluate the potential influence of race, we incorporated subjects from two cohorts representing four different races to re-perform the regression analyses. The results indicated that even after adjusting for race and other crucial confounding factors such as age, gender, and proteinuria, uL-selectin remained an independent predictor of high CI in the multivariate logistic regression analyses (**Table S12**).

**Figure 3:**
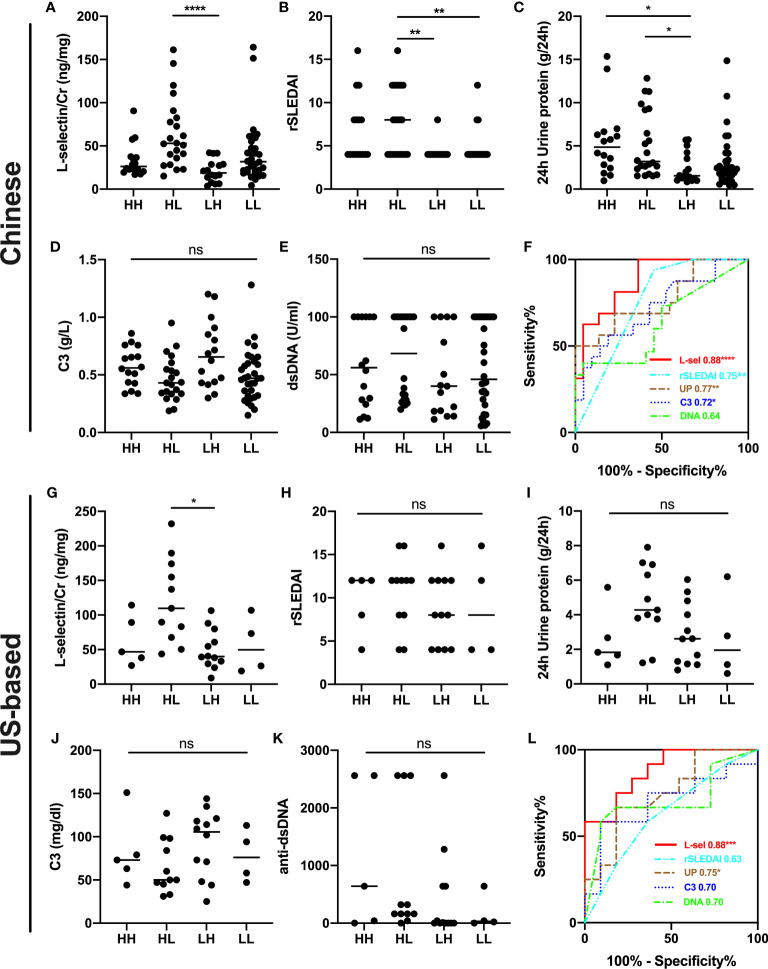
Subgroup analyses for combined AI+CI in the Chinese cohort **(A–F)** and US-based cohort **(G–L)**. uL-selectin levels **(A, G)**, rSLEDAI **(B, H),** 24h urine protein **(C, I)**, C3 levels **(D, J)** and anti-dsDNA levels **(E, K)** were shown in four subgroups of LN patients; Kruskal-Wallis test and Dunn’s multiple comparisons test. **(F, L)** ROC curve analyses were performed to discriminate HL subgroup from LH subgroup of LN patients. HH both high AI (AI>6) and high CI (CI>3); HL both high AI (AI>6) and low CI (CI ≤ 3); LH both low AI (AI ≤ 6) and high CI (CI>3); LL both low AI (AI ≤ 6) and low CI (CI ≤ 3); AI activity index; CI chronicity index; ns, no significance. *P<0.05, **P<0.01, ****P <0.0001.

In addition, biopsy-concurrent LN patients were divided into four subgroups with combined AI and CI. uL-selectin could significantly discriminate patients with both high AI (AI>6) and low CI (CI ≤ 3) (HL subgroup) from those with both low AI (AI ≤ 6) and high CI (CI>3) (LH subgroup) in both Chinese cohort and US-based cohort, which showed a better performance than 24h urine protein in differentiating these two subgroups by ROC curve analyses (**Figure 3**). In the Chinese cohort, rSLEDAI also could discriminate HL group from low AI group (LH group + LL group). In addition, other conventional markers such as C3 levels and anti-dsDNA levels showed no statistical differences in the four subgroups of the two cohorts.

### Changes in uL-selectin were associated with treatment response

**Figure 4:**
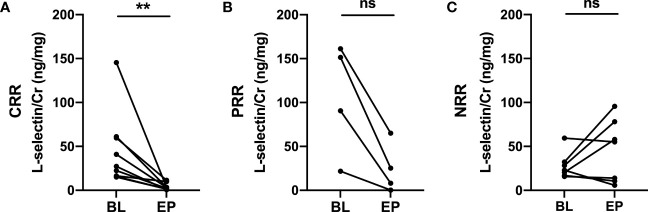
Urinary L-selectin levels in active LN patients at the endpoints of follow up compared with those at baseline. **(A)** Urinary L-selectin levels were decreased in the complete renal remission group (CRR) (n=9, P=0.0039) at the end of follow-up, while **(B)** it remained stable in the partial renal remission group (PRR) (n=4, P=0.125) and **(C)** in the no renal remission group (NRR) (n=7, P=0.578). BL, baseline; EP, endpoint; ns, no significance. Wilcoxon matched-paired signed rank test. **P ≤ 0.01.

Twenty SLE patients with active lupus nephritis in the Chinese cohort were followed up for at least 6 months. At the end of follow up, 13 patients achieved renal remission, 9 of whom achieved complete renal remission (CRR) and 4 patients achieved partial renal remission (PRR), while 7 patients had no renal remission (NRR). Importantly, uL-selectin significantly decreased in the complete renal remission group at the end of follow-up (p = 0.0039), while in the partial remission (p = 0.125) and no renal remission group (p = 0.578), uL-selectin displayed no differences (**Figures 4A-C**; **Table S13**).

## Discussion

Previous studies have shown that the serum levels of L-selectin were associated with several autoimmune diseases (11, 12). A recent study using array-based proteomics had identified uL-selectin as a novel biomarker for LN with good performance in reflecting disease activity (4). As a pre-requisite for eventual clinical use, here we test the biomarker potential of uL-selectin in two different laboratories in two nations, using multi-center, multi-ethnic cohorts. These studies have successfully validated uL-selectin as a promising biomarker for disease activity, renal histological changes and treatment response in LN. In the Chinese cohort, this urinary molecule was elevated exclusively in active LN patients (compared to other SLE and CKD patients) and showed better performance than conventional markers to discriminate active LN from active non-renal SLE. It also correlated with systemic and renal disease activity in LN. Importantly, while high uL-selectin was predictive of concurrent renal pathological activity, low uL-selectin emerged as an independent predictor of high CI, and could significantly discriminate patients with AI >6 and CI ≤3 from those with AI ≤ 3 and CI>3, showing similar patterns observed in both cohorts, and performing better than proteinuria. Furthermore, a longitudinal study demonstrated the potential role of uL-selectin in monitoring disease activity and treatment response in active LN. Comparable results were also observed in the US-based cohort comprised of Caucasian, African American and Hispanic subjects. The findings of significant increase of uL-selectin levels in active LN patients compared with healthy controls and its correlation with 24h proteinuria, serum C3, C4, ESR and AI were consistent across all four ethnicities. Possible reasons for the subtle difference noted between ethnicities or cohorts (e.g., correlation with CI) may relate to the inadequate sample size in some ethnic/racial groups and potential genetic heterogeneity (13).

Additional analyses of uL-selectin levels were also performed in 33 patients with CKD (**Table S14**). They had higher levels of uL-selectin compared to HC. No difference of uL-selectin levels was found among different types or stages of CKD. (**Figure S5**). Although uL-selectin levels in CKD patients were lower than those in active LN patients and comparable to those in active non-renal SLE patients, it indeed indicates that uL-selectin could be a more general biomarker of renal involvement, and not SLE-specific, which is in line with the results by Vanarsa et al. (4).

L-selectin, also called CD62L, is a type I transmembraenne cell adhesion molecule broadly expressed on neutrophils, monocytes and most circulating leukocytes. The sticky binding of this molecule to its ligands on endothelial cells or other leukocytes triggers cell adhesion and migration from blood vessels to sites of local inflammation. The process of rolling and transendothelial migration (TEM) activates inducible-shedding of the molecule on the cells and results in the release of soluble (s) L-selectin into body fluids (14). Like other cell adhesion molecules, L-selectin expressed on renal-infiltrating leukocytes may play a pathogenic role in renal tissue inflammation and disease progression in LN, and this warrants further mechanistic investigation.

Although uL-selectin levels were positively correlated with AI, the association of high uL-selectin levels with high AI did not attain statistical significance by univariate or multivariate logistic regression analyses after adjusting for 24h proteinuria, indicating the weak correlation might be caused by other confounding factors such as proteinuria and suggesting the potential influence of urinary leakage in active renal injury in interpreting urine biomarker levels (**Tables S15-17**). However, lower uL-selectin level was independently associated with high CI, which is an independent risk factor of poor prognosis in LN (15), even after adjusting for 24h proteinuria and eGFR, with similar patterns being observed in both cohorts. It is important to note that the relationship between uL-selectin levels and CI in LN may have important implications for predicting disease prognosis and guiding treatment decisions. However, further research may be necessary to better understand the underlying mechanisms driving this association and to determine the clinical utility of measuring uL-selectin levels in the context of LN.

Conventional markers such as proteinuria may not be informative in assessing pathological activity and chronicity in lupus nephritis. uL-selectin showed a better performance in discriminating patients with high AI and low CI from those with high CI and low AI in kidney than proteinuria, which was observed in both cohorts. The scRNAseq data suggested that urinary L-selectin is derived in large part from infiltrating B-cells, especially on activated and ISG-high B cells (4). B-cells are less involved in renal fibrosis than in active glomerulonephritis, therefore LN patients with higher activity and lower chronicity would have larger urinary L-selectin excretion compared to patients with higher chronicity and lower activity. High uL-selectin with high proteinuria may reflect high AI and low CI in kidney which needs aggressive treatment, while low uL-selectin may reflect high CI and low AI which should be treated with more caution even the proteinuria level is relatively high.

Extrapolating from the results of the current study, we envision the following utilities of uL-selectin in clinical practice. Firstly, monitoring uL-selectin levels can help objectively assess clinical disease activity in LN at each follow-up visit. Secondly, close monitoring of uL-selectin levels may help predict response to treatment in terms of complete renal remission although larger prospective studies are warranted to validate this.

The limitations of this study include several important aspects that need to be considered carefully when interpreting the results. One inherent limitation is that requires consideration pertains to the restricted size of the population under investigation. This constraint becomes particularly conspicuous when focusing on specific demographic subsets, namely the Caucasian and Hispanic cohorts, as well as the longitudinal cohort. Indeed, the issue of limited population size may notably impact the generalizability of the study’s findings. A broader cross-section of ethnic backgrounds within the cohort would have facilitated a more robust assessment of the relationships and trends explored in our research. Therefore, future endeavors should prioritize the inclusion of a more extensive multi-ethnic population and a prospective longitudinal cohort to further validate and strengthen the outcomes observed here. To mitigate these limitations, we employed statistical techniques to account for the constraints. We carefully performed a rigorous sample size calculation to ensure statistical power in relation to the specific objectives of this study. Despite the inherent limitations, the current sample size was determined to be adequate to detect the expected effect sizes within the context of our research questions. However, it remains imperative to acknowledge the potential impact of these limitations on the precision and applicability of our findings. Another limitation is that we cannot exclude the potential impact of medications used for clinical treatment on the levels of uL-selectin. Additionally, the absence of assessment of L-selectin levels in the blood and kidneys of LN patients limits our knowledge of the exact source of its origin in urine. Extended studies with larger sample sizes in both the cross-sectional and longitudinal cohorts, together with parallel assessment of competing biomarker candidates, are warranted before one can endorse the use of uL-selectin in routine clinical practice.

## Data availability statement

The original contributions presented in the study are included in the article/**Supplementary Material**. Further inquiries can be directed to the corresponding authors.

## Ethics statement

The ethics committee of Renji Hospital, Shanghai Jiao Tong University School of Medicine, Shanghai, China; The Institutional Review Boards of the University of Houston, JHU School of Medicine, and UTSW. The studies were conducted in accordance with the local legislation and institutional requirements. The human samples used in this study were acquired from primarily isolated as part of your previous study for which ethical approval was obtained. Written informed consent for participation was not required from the participants or the participants’ legal guardians/next of kin in accordance with the national legislation and institutional requirements.

## Author contributions

HD, CM, and ZZY designed the study and planned this work with YS, KV, and ZHY. YS, KV, and ZHY carried out the experiments, acquired the data, and drafted the manuscript. HD, YS, TZ, JW, and NS performed data analysis and interpretation of the results. TZ, JC, MD, and LZ contributed to sample processing and clinical data collection. LQ, QG, RS, and MP contributed to patient recruiting and follow-up. HD, CM, and NS reviewed and revised the manuscript. The manuscript was written through the contributions of all authors. All authors contributed to the article and approved the submitted version.
