# Corrigendum: Urine L-selectin reflects clinical and histological renal disease activity and treatment response in lupus nephritis across multi-ethnicity

**DOI:** 10.3389/fimmu.2023.1295153

**Published:** 2023-10-30

**Authors:** Yiwei Shen, Kamala Vanarsa, Zhihua Yin, Ting Zhang, Jessica Castillo, Min Dai, Linghua Zou, Ling Qin, Jieying Wang, Qiang Guo, Ramesh Saxena, Michelle Petri, Nan Shen, Zhizhong Ye, Chandra Mohan, Huihua Ding

**Affiliations:** ^1^Department of Rheumatology, Shanghai Institute of Rheumatology, Renji Hospital, Shanghai Jiao Tong University School of Medicine, Shanghai, China; ^2^Department of Biomedical Engineering, University of Houston, Houston, TX, United States; ^3^Department of Rheumatology, Shenzhen Futian Hospital for Rheumatic Diseases, Shenzhen, China; ^4^Department of Rheumatology, Joint Research Laboratory for Rheumatology of Shenzhen University Health Science Center and Shenzhen Futian Hospital for Rheumatic Diseases, Shenzhen, China; ^5^Division of Rheumatology, The Second Affiliated Hospital, Zhejiang University School of Medicine, Zhejiang, China; ^6^Department of Rehabilitation, Shenzhen Futian Hospital for Rheumatic Diseases, Shenzhen, China; ^7^Department of Nephrology, Shanghai Tenth People’s Hospital, Tongji University School of Medicine, Shanghai, China; ^8^Clinical Research Unit, Renji Hospital, Shanghai Jiao Tong University School of Medicine, Shanghai, China; ^9^Division of Nephrology, University of Texas Southwestern Medical Center, Dallas, TX, United States; ^10^Division of Rheumatology, Johns Hopkins University School of Medicine, Baltimore, MD, United States; ^11^State Key Laboratory of Oncogenes and Related Genes, Shanghai Cancer Institute, Renji Hospital, Shanghai Jiao Tong University School of Medicine (SJTUSM), Shanghai, China; ^12^Center for Autoimmune Genomics and Etiology, Cincinnati Children’s Hospital Medical Center, Cincinnati, OH, United States; ^13^Department of Pediatrics, University of Cincinnati College of Medicine, Cincinnati, OH, United States

**Keywords:** lupus nephritis, L-selectin, urinary biomarker, renal histopathology, treatment response

In the published article, there was an error. In the results section of the abstract, there is an error in the statement.

A correction has been made to the Abstract, Results section, Sentence 2. This sentence previously stated:

“uL-selectin positively correlated with global and renal disease activities as well as histological activity index and chronicity index (CI).”

The corrected sentence appears below:

“uL-selectin positively correlated with global and renal disease activities and was significantly associated with histological activity index and chronicity index (CI).”

The authors apologize for this error and state that this does not change the scientific conclusions of the article in any way. The original article has been updated.

