# Supplementary material for: Urine L-selectin reflects clinical and histological renal disease activity and treatment response in lupus nephritis across multi-ethnicity
Source: Front Immunol. 2023 Aug 31;14:1200167. doi: 10.3389/fimmu.2023.1200167 (PMC10500131; doi:10.3389/fimmu.2023.1200167)
Supplement: Supplementary file 1 [file DataSheet_1.pdf]

SUPPLEMENTARY MATERIALS

SUPPLEMENTARY METHODS

Renal response to therapy at the end of follow-up

- Patients who achieve Complete Renal Remission (CRR) met the following criteria: 1) proteinuria < 0.5g/24h; 2) stabilization or improvement in kidney function (± 15% of baseline).
- Patients who achieve Partial Renal Remission (PRR) met the following criteria:
  1) reduction in proteinuria by at least 50% and to < 3g/24h;
  2) stabilization or improvement in kidney function (± 15% of baseline).
- Patients who achieved neither CRR nor PRR are defined as No Renal Remission (NRR).

SUPPLEMENTARY TABLES

Supplementary Table 1. Demographic and clinical characteristics of study subjects from the Chinese cohort (n=255).

Supplementary Table 2. Demographic and clinical characteristics with Caucasian patients of the US-based cohort (n=34).

Supplementary Table 3. Demographic and clinical characteristics with African American patients of the US-based cohort (n=114).

Supplementary Table 4. Demographic and clinical characteristics with Hispanic patients of the US-based cohort (n=71).

Supplementary Table 5. Demographic and clinical characteristics of active LN patients with concurrent renal biopsies in the US-based cohort (n=32).

Supplementary Table 6. Correlation analysis between urinary L-selectin levels and laboratory parameters in SLE patients (n=195) from the Chinese cohort.

Supplementary Table 7. Correlation analysis of urinary L-selectin with clinical features of SLE patients (n=195) from the Chinese cohort within one month before recruitment.

Supplementary Table 8. Correlation analysis between urinary L-selectin levels and laboratory parameters of SLE patients in the US-based cohort.

Supplementary Table 9. Correlation of urinary L-selectin and conventional metrics with renal pathology activity and chronicity indices in biopsy-concurrent LN patients in the Chinese cohort (n=87).

Supplementary Table 10. Univariate logistic regression analyses for high CI (CI>3) in the Chinese cohort.

Supplementary Table 11. Multivariate logistic regression analyses for high CI (CI>3) in the Chinese cohort.

Supplementary Table 12. Multivariate logistic regression analyses for high CI (CI>3) in the two cohorts (n=119).

Supplementary Table 13. Comparison of remission and non-remission groups in the follow-up cohort of the Chinese cohort (n=18).

Supplementary Table 14. Demographics and clinical characteristics of patients with chronic kidney diseases (n=33).

Supplementary Table 15. Univariate logistic regression analyses for high AI (AI>6) in the Chinese cohort.

Supplementary Table 16. Multivariate logistic regression analyses for high AI (AI>6) in the Chinese cohort.

Supplementary Table 17. Multivariate logistic regression analyses for high AI (AI>6) in the two cohorts (n=119).

SUPPLEMENTARY FIGURES

Supplementary Figure 1. Flow diagram of the study.

Supplementary Figure 2. uL-selectin levels in LN patients with concurrent renal biopsies parsed by pathology class in the Chinese cohort.

Supplementary Figure 3. Subgroup analyses for AI and CI in the Chinese cohort.

Supplementary Figure 4. Correlation analyses and subgroup analyses for AI and CI in the US-based cohort.

Supplementary Figure 5. Subgroup analyses of CKD patients in the Chinese cohort.

Table S1. Demographic and clinical characteristics of study subjects from the Chinese cohort (n=255).

	SLE				CKD	HC	P-value[#]
	aLN	aNR	iLN	iNR			
**N**	87	57	25	26	33	27	
**Age (years), (mean ± SD)**	33.9 ± 12.4	31.1 ± 12.2	36.0 ± 8.6	34.1 ± 11.5	59.0 ± 11.6	35.2 ± 10.2	0.106
**Female, n (%)**	82 (94.3)	54 (94.8)	23 (92.0)	25 (100.0)	13 (39.4)	22 (81.5)	0.593
**SLE duration (months)[&]**	36.0 (101.0)	3.0 (11.0)	27.4 (54.7)	17.7 (120.0)	24.0 (55.0)	N/A	<0.001
**Clinical characteristics, n (%)[§]**							
Fever	19 (21.8)	23 (40.4)	0 (0)	4 (15.4)	N/A	N/A	<0.001
Lymphadenopathy	21 (24.1)	11 (19.3)	0 (0)	1 (3.9)	N/A	N/A	0.008
Malar rash	26 (29.9)	29 (50.9)	0 (0)	2 (7.7)	N/A	N/A	<0.001
Mucosal ulceration	2 (2.2)	6 (10.5)	0 (0)	0 (0)	N/A	N/A	0.031
Alopecia	16 (18.4)	17 (29.8)	0 (0)	0 (0)	N/A	N/A	<0.001
Vasculitis	2 (2.2)	3 (5.3)	0 (0)	2 (7.7)	N/A	N/A	0.380
Dry eyes and/or mouth	2 (2.2)	7 (12.3)	0 (0)	1 (3.9)	N/A	N/A	0.031
Raynaud's phenomenon	7 (8.1)	5 (8.8)	0 (0)	0 (0)	N/A	N/A	0.207
NPSLE	0 (0)	0 (0)	0 (0)	0 (0)	N/A	N/A	—
Myositis	1 (1.2)	0 (0)	0 (0)	0 (0)	N/A	N/A	0.742
Arthralgia/arthritis	21 (24.1)	41 (71.9)	0 (0)	1 (3.9)	N/A	N/A	<0.001
Endocarditis/Pleural effusion	29 (33.3)	6 (10.5)	0 (0)	0 (0)	N/A	N/A	<0.001
PAH	3 (3.5)	0 (0)	0 (0)	0 (0)	N/A	N/A	0.286
ILD	2 (2.3)	0 (0)	0 (0)	0 (0)	N/A	N/A	0.474
**Clinical assessment, median (IQR)**							
SLEDAI	10 (6)	11 (3.5)	4 (2)	4 (0.5)	N/A	N/A	<0.001
rSLEDAI	4 (4)	0 (0)	0 (0)	0 (0)	N/A	N/A	<0.001
SLICC RAS	8 (6)	N/A	N/A	N/A	N/A	N/A	—
**Laboratory tests, median (IQR)**							
24-h urine protein (g/24h)	2.65 (4.0)	0.13 (0.2)	0.14 (0.2)	0.10 (0.1)	2.88 (4.9)	N/A	<0.001
anti-dsDNA titer (IU/mL)	46.7 (74.7)	100 (60.7)	22.3 (30.1)	38.9 (87.4)	N/A	N/A	<0.001
ANA titer	640 (960)	1280 (640)	320 (160)	640 (960)	N/A	N/A	<0.001
C3 (g/L)	0.48 (0.3)	0.52 (0.3)	0.85 (0.3)	0.77 (0.4)	N/A	N/A	<0.001
C4 (g/L)	0.08 (0.06)	0.08 (0.08)	0.16 (0.08)	0.14 (0.11)	N/A	N/A	<0.001
CH50 (U/mL)	25.4 (19.6)	27.4 (29.0)	44.2 (17.3)	46.9 (34.1)	N/A	N/A	<0.001
ESR (mm/1h)	37.0 (31.2)	44.5 (37.8)	14.0 (14.0)	36.0 (33.5)	N/A	N/A	<0.001
CRP (mg/L)	3.1 (1.9)	3.1 (6.2)	1.5 (2.8)	3.1 (10.3)	N/A	N/A	<0.001
eGFR-EPI (ml/min/1.73m^2)[$]	110 (62.3)	142 (51.1)	108 (46.2)	146 (46.5)	62 (50.1)	N/A	<0.001
Scr (umol/L)	58 (33)	49 (14)	59 (21)	47 (14)	109 (106)	N/A	<0.001
**Renal pathology (ISN/RPS classification), n (%)**							
Class II	1 (1.2)	N/A	N/A	N/A	N/A	N/A	—
Class III	11 (12.6)	N/A	N/A	N/A	N/A	N/A	—
Class IV	31 (35.6)	N/A	N/A	N/A	N/A	N/A	—
Class V	15 (17.2)	N/A	N/A	N/A	N/A	N/A	—
Class III + V	15 (17.2)	N/A	N/A	N/A	N/A	N/A	—

Class IV + V	14 (16.1)	N/A	N/A	N/A	N/A	N/A	—
**Glucocorticoid and immunosuppressants, n (%)***							
Prednisone	72 (82.8)	33 (57.9)	21 (76.9)	20 (76.9)	4 (12.1)	N/A	0.008
Dose, mg/day, median (IQR)	22.5 (40.6)	30.0 (30.0)	10.0 (11.3)	20.0 (37.5)	N/A	N/A	0.003
Hydroxychloroquine	42 (48.3)	23 (40.4)	16 (6)	14 (53.9)	0 (0)	N/A	0.226
Tacrolimus	7 (8.05)	1 (1.75)	3 (12)	0 (0)	0 (0)	N/A	0.197
Leflunomide	8 (9.20)	0 (0)	1 (4)	0 (0)	0 (0)	N/A	0.085
Azathioprine	5 (5.75)	2 (3.51)	6 (24)	0 (0)	0 (0)	N/A	0.005
Thalidomide	1 (1.2)	1 (1.8)	1 (4)	2 (7.7)	0 (0)	N/A	0.433
Cyclophosphamide	7 (8.1)	1 (1.8)	3 (12)	2 (7.7)	4 (12.1)	N/A	0.453
Tripterygium Glucosides	4 (4.6)	0 (0)	0 (0)	0 (0)	2 (6.1)	N/A	0.280
Cyclosporin	3 (3.5)	0 (0)	1 (4)	1 (3.9)	0 (0)	N/A	0.708
Methotrexate	2 (2.3)	1 (1.8)	1 (4)	1 (3.9)	0 (0)	N/A	0.967
Mycophenolate mofetil	17 (19.5)	1 (1.8)	2 (8)	2 (7.7)	0 (0)	N/A	0.020
**Comorbidity, n (%)**							
RA	0 (0)	2 (3.5)	0 (0)	0 (0)	0 (0)	N/A	0.180
SS	4 (4.6)	2 (3.5)	0 (0)	0 (0)	0 (0)	N/A	0.507
APS	4 (4.6)	0 (0)	0 (0)	0 (0)	0 (0)	N/A	0.167
Hyperuricemia	0 (0)	0 (0)	0 (0)	0 (0)	13 (39.4)	N/A	—
AVN of femoral head	1 (1.15)	1 (1.8)	1 (4)	1 (3.9)	0 (0)	N/A	0.737
Hypertension	22 (25.3)	2 (3.5)	3 (12.0)	3 (11.5)	25 (75.8)	N/A	0.004
Diabetes mellitus	0 (0)	1 (1.8)	0 (0)	1 (3.9)	14 (42.4)	N/A	0.321
Hyperlipidemia	2 (2.3)	0 (0)	0 (0)	0 (0)	11 (33.3)	N/A	0.474
**Urine L-selectin/Cr (ng/mg), median (IQR)**							
	31.4 (34.2)	4.0 (7.6)	1.1 (3.3)	2.7 (4.1)	6.7 (9.1)	0.4 (0.8)	<0.0001

Data were presented as number (percentage), average ± standard deviation, or median (IQR). **aLN** active lupus nephritis; **aNR** active SLE without renal involvement; **iLN** inactive lupus nephritis; **iNR** inactive SLE without renal involvement; **CKD** chronic kidney disease; **HC** healthy control; **NPSLE** neuropsychiatric systemic lupus erythematosus; **PAH** pulmonary arterial hypertension; **ILD** interstitial lung disease; **SLEDAI** Systemic Lupus Erythematosus Disease Activity Index; **rSLEDAI** renal SLEDAI; **SLICC RAS** SLICC renal activity score; **ANA** anti-nuclear antibody; **C3** complement 3; **C4** complement 4; **CH50** 50% hemolytic unit of complement; **ESR** erythrocyte sedimentation rate; **CRP** C-reactive protein; **eGFR** estimated glomerular filtration rate; **Scr** serum creatinine; **ISN/RPS classification** International Society of Nephrology/Renal Pathology Society classification; **RA** rheumatoid arthritis; **SS** Sjogren's syndrome; **APS** antiphospholipid syndrome; **AVN** avascular necrosis; **SD** standard deviation; **IQR** interquartile range. [&] The results of SLE disease duration were shown by median (IQR); [#] P values were shown for the comparison among subgroups of SLE patients by one-way ANOVA (Non-parametric) or Chi-Squared Test/Fisher's exact test; [§] Clinical characteristics of patients appeared within one month before entrance into the cohort; [$]Serum estimated glomerular filtration rate (eGFR) was calculated by 2021 CKD-EPI Creatinine equation: 142 x $(Scr/A)^{B}$ x 0.9938^{age} x (1.012 if female), where A and B are the following: Female if Scr≤0.7, then A=0.7, B=-0.241; if Scr >0.7, then A=0.7, B=-1.2; Male if Scr≤0.9, then A=0.9, B=-0.302; if Scr>0.9, then A=0.9, B=-1.2; [*]Medication information of patients was collected within one month before entrance into the cohort.

Table S2. Demographic and clinical characteristics with Caucasian patients of the US-based cohort (n=34).

	**aLN (N=17)**	**aNR (N=3)**	**iLN (N=3)**	**HC (N=11)**
**Age (years), mean ± SD**	35.7 ± 12.4	50.3 ± 17.7	47.0 ± 19.3	32.0 ± 8.5
**Female, n (%)**	17 (100)	3 (100)	2 (66.7)	5 (45.5)
**Clinical characteristics, n (%)**				
Joints	4 (23.5)	0 (0)	0 (0)	NA
Mucocutaneous	3 (17.6)	3 (100)	0 (0)	NA
Hematological	2 (11.8)	0 (0)	1 (33.3)	NA
Neurological	0 (0)	0 (0)	0 (0)	NA
**Clinical assessment, median (IQR)***				
SLEDAI	8 (6)	6 (NA)	0 (NA)	NA
rSLEDAI	4 (4)	0 (NA)	0 (NA)	NA
**Laboratory measurement, median (IQR)***				
24-h urine protein (g/24h)	1.4 (4.4)	0.07 (NA)	0.05 (NA)	NA
anti-dsDNA titer (IU/mL)	320 (560)	80 (NA)	80 (NA)	NA
C3 (mg/dl)	79 (48)	47 (NA)	128 (NA)	NA
C4 (mg/dl)	9 (4)	9 (NA)	16 (NA)	NA
Scr (mg/dl)	0.7 (0.6)	0.8 (NA)	2.8 (NA)	NA
**Medications, n (%)**				
Prednisone	17 (100)	2 (66.7)	1 (33.3)	NA
Hydroxychloroquine	10 (58.8)	3 (100)	3 (100)	NA
Mycophenolate mofetil	15 (88.2)	0 (0)	0 (0)	NA
Azathioprine	1 (5.9)	0 (0)	1 (33.3)	NA
Cyclosporin	1 (5.9)	0 (0)	0 (0)	NA
Tacrolimus	0 (0)	0 (0)	0 (0)	NA
Cyclophosphamide	0 (0)	1 (33.3)	0 (0)	NA
Methotrexate	0 (0)	0 (0)	0 (0)	NA
**Urine L-selectin/Cr (ng/mg), median (IQR)**				
	154 (316)	3 (NA)	8 (NA)	12 (21)

Data were presented as number (percentage), average ± standard deviation, or median (IQR). **aLN** active lupus nephritis; **aNR** active SLE without renal involvement; **iLN** inactive lupus nephritis; **HC** healthy control; **SD** standard deviation; **IQR** interquartile range; * Data were not shown by IQR in aNR and iLN groups due to the small size.

Table S3. Demographic and clinical characteristics with African American patients of the US-based cohort (n=114).

	**aLN (N=59)**	**aNR (N=14)**	**iLN (N=20)**	**HC (N=21)**
**Age (years), mean ± SD**	29.9 ± 7.1	39.0 ± 10.0	32.5 ± 5.5	34.2 ± 7.0
**Female, n (%)**	58 (98.3)	14 (100)	20 (100)	16 (76.2)
**Clinical characteristics, n (%)**				
Joints	9 (15.3)	6 (42.9)	0 (0)	NA
Mucocutaneous	15 (25.4)	12 (85.7)	0 (0)	NA
Hematological	9 (15.3)	1 (7.1)	1 (5.0)	NA
Neurological	0 (0)	0 (0)	0 (0)	NA
**Clinical assessment, median (IQR)**				
SLEDAI	8 (4)	6 (2.5)	0 (2)	NA
rSLEDAI	4 (4)	0 (0)	0 (0)	NA
**Laboratory measurement, median (IQR)**				
24-h urine protein (g/24h)	1.7 (4.5)	0.1 (0.2)	0.3 (0.3)	NA
anti-dsDNA titer (IU/mL)	28 (320)	80 (640)	0 (3)	NA
C3 (mg/dl)	84 (46)	93 (51)	113 (40)	NA
C4 (mg/dl)	15 (15)	15 (8)	21 (14)	NA
Scr (mg/dl)	1.0 (0.4)	0.8 (0.3)	0.9 (0.5)	NA
**Medications, n (%)**				
Prednisone	50 (84.7)	6 (42.9)	12 (60.0)	NA
Hydroxychloroquine	33 (55.9)	11 (78.6)	14 (70.0)	NA
Mycophenolate mofetil	35 (59.3)	7 (50.0)	9 (45.0)	NA
Azathioprine	5 (8.5)	0 (0)	1 (5.0)	NA
Cyclosporin	1 (1.7)	0 (0)	0 (0)	NA
Tacrolimus	0 (0)	1 (7.1)	0 (0)	NA
Cyclophosphamide	3 (5.1)	0 (0)	0 (0)	NA
Methotrexate	0 (0)	1 (7.1)	0 (0)	NA
**Urine L-selectin/Cr (ng/mg), median (IQR)**				
	19 (42)	1 (3)	2 (7)	2 (3)

Data were presented as number (percentage), average ± standard deviation, or median (IQR). **aLN** active lupus nephritis; **aNR** active SLE without renal involvement; **iLN** inactive lupus nephritis; **HC** healthy control; **SD** standard deviation; **IQR** interquartile range.

Table S4. Demographic and clinical characteristics with Hispanic patients of the US-based cohort (n=71).

	**aLN (N=45)**	**iLN (N=5)**	**HC (N=21)**
**Age (years), mean ± SD**	32.6 ± 10.2	31.0 ± 9.2	30.9 ± 8.2
**Female, n (%)**	41 (91.1)	5 (100)	17 (81.0)
**Clinical characteristics, n (%)**			
Joints	8 (17.8)	0 (0)	NA
Mucocutaneous	12 (26.7)	0 (0)	NA
Hematological	13 (28.9)	0 (0)	NA
Neurological	1 (2.2)	0 (0)	NA
**Clinical assessment, median (IQR)**			
SLEDAI	12 (8)	0 (2)	NA
rSLEDAI	8 (8)	0 (0)	NA
**Laboratory measurement, median (IQR)**			
24-h urine protein (g/24h)	2.4 (2.6)	0.2 (0.3)	NA
anti-dsDNA titer (IU/mL)	160 (1920)	0 (0)	NA
C3 (mg/dl)	84 (60)	98 (35)	NA
C4 (mg/dl)	15 (13)	25 (6)	NA
Scr (mg/dl)	0.9 (1.0)	0.9 (1.7)	NA
**Medications, n (%)**			
Prednisone	39 (86.7)	2 (40.0)	NA
Hydroxychloroquine	26 (57.8)	3 (60.0)	NA
Mycophenolate mofetil	19 (42.2)	2 (40.0)	NA
Azathioprine	5 (11.1)	1 (20.0)	NA
Cyclosporin	0 (0)	1 (20.0)	NA
Tacrolimus	0 (0)	0 (0)	NA
Cyclophosphamide	4 (8.9)	0 (0)	NA
Methotrexate	0 (0)	0 (0)	NA
**Urine L-selectin/Cr (ng/mg), median (IQR)**			
	38.4 (67.6)	9.6 (21.7)	0.8 (3.9)

Data were presented as number (percentage), average ± standard deviation, or median (IQR). **aLN** active lupus nephritis; **aNR** active SLE without renal involvement; **iLN** inactive lupus nephritis; **HC** healthy control; **SD** standard deviation; **IQR** interquartile range.

Table S5. Demographic and clinical characteristics of active LN patients with concurrent renal biopsies in the US-based cohort (n=32).

	**Concurrent-biopsy LN (N=32)**
**Race, n (%)**	
Caucasian	5 (15.6)
African American	10 (31.3)
Hispanic	17 (53.1)
**Age (years), mean ± SD**	32 ± 9.3
**Female, n (%)**	30 (93.8)
**Clinical assessment, median (IQR)**	
SLEDAI	11 (8)
rSLEDAI	8 (8)
**Renal pathology (ISN/RPS classification)**	
Class I	0 (0)
Class II	1 (3.1)
Class III	10 (31.3)
Class IV	7 (21.9)
Class V	2 (6.3)
Class III+V	8 (25.0)
Class IV+V	4 (12.5)
**Activity Index, median (IQR)**	6 (8)
**Chronicity Index, median (IQR)**	4 (4)
**Medications, n (%)**	
Prednisone	32 (100.0)
Cyclophosphamide	1 (3.1)
Mycophenolate mofetil	23 (71.9)
Azathioprine	3 (9.4)
Cyclosporin	0 (0)
**Urine L-selectin/Cr (ng/mg), median (IQR)**	
	52.5 (61.6)

Data were presented as number (percentage), average ± standard deviation, or median (IQR). **SD** standard deviation; **IQR** interquartile range.

Table S6. The correlation analysis between urinary L-selectin levels and laboratory parameters in SLE patients (n=195) from the Chinese cohort.

	r	95% CI	p-value
**24h Upro (g/24h)**	0.82	(0.76, 0.86)	**<0.0001**
**Cr (mg/dl)**	0.17	(0.02, 0.31)	**0.021**
**eGFR (ml/min/1.73m^2)**	-0.16	(-0.30, -0.02)	**0.026**
**anti-dsDNA (IU/mL)**	0.11	(-0.03, 0.26)	0.121
**ANA titers**	-0.02	(-0.18, 0.14)	0.789
**C3 (g/L)**	-0.36	(-0.48, -0.22)	**<0.0001**
**C4 (g/L)**	-0.24	(-0.37, -0.09)	**0.0011**
**CH50 (U/ml)**	-0.38	(-0.50, -0.24)	**<0.0001**
**ESR (mm/1h)**	0.29	(0.15, 0.42)	**<0.0001**
**CRP (mg/L）**	0.14	(-0.01, 0.29)	0.0596
**IgG（g/L）**	-0.18	(-0.33, -0.02)	**0.0216**
**IgA（g/L）**	-0.04	(-0.20, 0.12)	0.635
**IgM（g/L）**	-0.12	(-0.27, 0.05)	0.146
**U1RNP**	0.04	(-0.13, 0.20)	0.661
**SSA/Ro52**	-0.08	(-0.24, 0.08)	0.330
**SSA/Ro60**	-0.12	(-0.27, 0.04)	0.138
**Anti-RIbP**	0.06	(-0.10, 0.22)	0.425
**CENP-B**	-0.02	(-0.18, 0.14)	0.827
**Anti-PM/Scl**	-0.003	(-0.16, 0.16)	0.966
**PCNA**	-0.07	(-0.23, 0.09)	0.365
**ACA-IgA (APL/ml)**	-0.05	(-0.21, 0.12)	0.569
**ACA-IgM (MPL/ml)**	-0.10	(-0.25, 0.07)	0.234
**ACA-IgG (GPL/ml)**	0.003	(-0.16, 0.17)	0.967
**Sm**	0.07	(-0.09, 0.23)	0.389
**SSB**	-0.10	(-0.26, 0.06)	0.207
**Jo-1**	-0.007	(-0.17, 0.16)	0.936
**Scl-70**	0.06	(-0.10, 0.22)	0.422
**AHA**	0.12	(-0.04, 0.28)	0.123
**AnuA**	0.14	(-0.02, 0.29)	0.081
**Beta2GP1 IgG**	-0.06	(-0.24, 0.12)	0.475
**Beta2GP1 IgM**	-0.10	(-0.27, 0.08)	0.249
**WBC**	0.02	(-0.12, 0.16)	0.764
**Hemoglobin**	-0.36	(-0.48, -0.23)	**<0.0001**
**PLT**	-0.16	(-0.30, -0.02)	**0.025**
**Uric acid**	0.39	(0.25, 0.52)	**<0.0001**
**TC**	0.38	(0.21, 0.53)	**<0.0001**
**TG**	0.38	(0.21, 0.52)	**<0.0001**
**HDL-C**	0.13	(-0.06, 0.30)	0.163
**LDL-C**	0.36	(0.19, 0.51)	**<0.0001**
**Apolipoprotein A1**	0.13	(-0.06, 0.31)	0.177
**Apolipoprotein B**	0.40	(0.22, 0.55)	**<0.0001**

**CK**	0.01	(-0.15, 0.18)	0.865

Anti-RIbP anti-ribosomal P protein antibody; **CENP-B** anti-centromere protein B antibody; **Anti-PM/Scl** anti-polymyositis scleroderma antibody; **PCNA** proliferating cell nuclear antigen; **ACA-IgA** anticardiolipin antibody IgA; **ACA-IgM** anticardiolipin antibody IgM; **ACA-IgG** anticardiolipin antibody IgG; **Sm** anti-Smith antibody; **SSB** anti-SSB antibody; **Jo-1** anti-Jo-1 antibody; **Scl-70** anti-Scl-70 antibody; **AHA** anti-histone antibody; **AnuA** anti-nucleosome antibody; **Beta2GP1 IgG** anti-Beta2GP1 IgG antibody; **Beta2GP1 IgM** anti-Beta2GP1 IgM antibody. **WBC** white blood cell; **PLT** platelet; **TC** total cholesterol; **TG** triglyceride; **HDL-C** high-density lipoprotein cholesterol; **LDL-C** low-density lipoprotein cholesterol; **CK** creatine kinase; **r**, Spearman's correlation coefficient.

Table S7. Correlation analysis of urinary L-selectin with clinical features of SLE patients (n=195) from the Chinese cohort within one month before recruitment.

**Groups**[&]	**Subgroups**	**N=195**	**L-selectin/Cr (ng/mg)**	
			**Median (IQR)**	**p-value**[%]
**Treatment**	Yes	155	10.2 (2.35-28.7)	0.764
	No	40	6.36 (2.45-26.0)	
**Fever**	Yes	46	12.8 (3.04-41.2)	0.145
	No	149	9.36 (1.51-26.7)	
**Lymphadenopathy**	Yes	33	35.4 (5.62-53.3)	**<0.001**
	No	162	7.59 (1.48-23.4)	
**Rash**	Yes	57	13.5 (3.48-35.1)	0.172
	No	138	8.03 (1.89-27.2)	
**Alopecia**	Yes	33	15.2 (3.38-35.4)	0.264
	No	162	9.43 (2.09-26.3)	
**Raynaud's**	Yes	12	22.2 (8.94-60.6)	**0.047**
	No	183	8.97 (2.00-28.3)	
**Serositis**	Yes	35	41.60 (19.4-70.7)	**<0.001**
	No	160	6.59 (1.50-21.6)	
**Polyarticular**	Yes	63	8.40 (2.00-37.8)	0.779
	No	132	11.6 (2.67-28.3)	
**Mouth ulcer**	Yes	8	12.1 (2.59-19.7)	0.804
	No	187	9.51 (2.35-28.7)	
**Vasculitis**	Yes	7	10.91 (6.93-38.7)	0.547
	No	188	9.70 (2.18-28.4)	

[&] Patients are from four subgroups of SLE patients. [%] P-value was obtained by Mann-Whitney U test.

Table S8. Correlation analysis between urinary L-selectin levels and laboratory parameters of SLE patients in the US-based cohort.

	**24h Upro**	**sCr**	**dsDNA**	**C3**	**C4**	**ESR**	**ANA titer**
**r**	0.586	-0.082	0.330	-0.286	-0.296	-0.250	-0.232
**P**	<0.001	0.352	0.001	0.001	0.001	0.015	0.201
**Sig.**	***	ns	***	***	***	*	ns

24hUpro 24h urine protein; **sCr** serum creatinine; **dsDNA** anti-dsDNA antibody; **r**, Spearman's correlation coefficient; **Sig.** significance.

Table S9. Correlation of urinary L-selectin and conventional metrics with renal pathology activity and chronicity indices in biopsy-concurrent LN patients in the Chinese cohort (n=87).

	SELL	C3	dsDNA	Upro
**Activity Index (AI)**	0.335**	-0.192	0.168	0.356**
Endocapillary hypercellularity	0.329**	-0.277**	0.157	0.317**
Neutrophils/karyorrhexis	0.179	-0.044	-0.135	0.160
Cellular/ fibrocellular crescents	0.121	-0.032	0.151	0.330**
Fibrinoid necrosis	0.215*	-0.098	0.214*	0.087
Hyaline deposits	0.416**	-0.428**	0.267*	0.132
Interstitial inflammation	-0.275**	0.158	-0.041	0.067
**Chronicity Index (CI)**	-0.305**	0.270*	-0.126	0.044
Glomerulosclerosis	-0.218*	0.228*	-0.107	0.106
Fibrous crescent	-0.280**	0.241*	-0.201	-0.070
Interstitial fibrosis	-0.236*	0.080	-0.096	0.035
Tubular atrophy	-0.216*	0.229*	-0.077	0.027

SELL urinary L-selectin/Cr; **Upro** 24h urine protein. **r** Spearman's correlation analysis. *P<0.05, **P<0.01

Table S10. Univariate logistic regression analyses for high CI (CI>3) in the Chinese cohort.

**Variables**	**Odds Ratio (95%CI)**	**P-value**
**Age, years**	1.04 (1.00-1.08)	**0.047**
**Gender**		0.878
Male	1 (reference)	
Female	0.87 (0.14-5.48)	
**SLE duration, years**	1.00 (1.00-1.01)	0.159
**LN duration, months**	1.00 (0.99-1.01)	0.650
**Low C3 and/or C4**		0.138
No	1 (reference)	
Yes	0.26 (0.05-1.53)	
**SLEDAI**	0.86 (0.76-0.97)	**0.014**
**rSLEDAI**	0.97 (0.83-1.11)	0.642
**anti-dsDNA, IU/mL**	1.00 (0.98-1.01)	0.455
**24h urine protein, g/24h**	1.00 (0.88-1.14)	0.979
**eGFR, ml/min/1.73m^2**	0.98 (0.96-0.99)	**<0.001**
**uL-selectin/Cr, ng/mg**	0.96 (0.94-0.99)	**0.005**

C3 complement 3; **C4** complement 4**; SLEDAI** Systemic Lupus Erythematosus Disease Activity Index; **rSLEDAI** renal SLEDAI; **anti-dsDNA** anti-dsDNA antibody; **eGFR** estimated glomerular filtration rate; **uL-selectin/Cr** urinary L-selectin levels adjusted by creatinine.

Table S11. Multivariate logistic regression analyses for high CI (CI>3) in the Chinese cohort.

**Models**		**Unadjusted**		**Adjusted**[&]		**LR test**[#]	**AIC**[*]
	**Parameter**	**OR (95% CI)**	**P-value**	**OR (95% CI)**	**P-value**	**P-value**	
**Model 1**						**M 1' Vs M 1**	107.9
eGFR+	eGFR	0.97 (0.96-0.99)	<0.001	0.97 (0.96-0.99)	<0.001	<0.001	
proteinuria	Proteinuria	0.91 (0.78-1.05)	0.207	0.91 (0.76-1.08)	0.275		
**Model 1'**							97.0
eGFR+	eGFR	0.98 (0.96-0.99)	0.002	0.98 (0.96-0.99)	0.005		
proteinuria+	Proteinuria	1.11 (0.91-1.36)	0.300	1.23 (0.94-1.60)	0.127		
uL-selectin/Cr	uL-selectin/Cr	0.96 (0.93-0.99)	0.008	0.95 (0.92-0.98)	0.005		
**Model 2**						**M 2' Vs M 2**	116.3
SLEDAI+	SLEDAI	0.84 (0.74-0.96)	0.010	0.84 (0.73-0.96)	0.014	<0.001	
proteinuria	Proteinuria	1.07 (0.93-1.23)	0.347	1.09 (0.91-1.31)	0.349		
**Model 2'**							104.4
SLEDAI+	SLEDAI	0.92 (0.79-1.06)	0.241	0.91 (0.77-1.07)	0.240		
proteinuria+	Proteinuria	1.25 (1.03-1.51)	0.022	1.42 (1.10-1.83)	0.006		
uL-selectin/Cr	uL-selectin/Cr	0.95 (0.92-0.99)	0.007	0.95 (0.92-0.98)	0.004		
**Model 3**						**M 3' Vs M 3**	100.7
eGFR+	eGFR	0.97 (0.96-0.99)	<0.001	0.97 (0.96-0.99)	<0.001	0.008	
SLEDAI+	SLEDAI	0.83 (0.72-0.96)	0.011	0.80 (0.67-0.95)	0.010		
proteinuria	Proteinuria	0.98 (0.84-1.15)	0.829	1.01 (0.83-1.23)	0.907		
**Model 3'**							95.8
eGFR+	eGFR	0.98 (0.96-0.99)	0.001	0.98 (0.96-0.99)	0.003		
SLEDAI+	SLEDAI	0.88 (0.75-1.04)	0.129	0.85 (0.71-1.03)	0.100		
proteinuria+	Proteinuria	1.12 (0.92-1.38)	0.251	1.23 (0.96-1.65)	0.100		
uL-selectin/Cr	uL-selectin/Cr	0.97 (0.94-0.99)	0.040	0.96 (0.93-0.99)	0.028		

OR odds ratio; **95% CI** 95% confidence interval; **LR test** likelihood ratio test; **eGFR** estimated glomerular filtration rate; **uL-selectin/Cr** urinary L-selectin levels adjusted by creatinine; **SLEDAI** Systemic Lupus Erythematosus Disease Activity Index;

[&]All models were adjusted for age, gender, SLE disease duration and LN disease duration; [#]LR tests were performed in two adjusted models; [*]AIC values were calculated in adjusted models, respectively.

Table S12. Multivariate logistic regression analyses for high CI (CI>3) in the two cohorts (n=119).

Models		Unadjusted		Adjusted[&]		LR test[#]	AIC[*]
	Parameter	OR (95% CI)	P-value	OR (95% CI)	P-value	P-value	
**Model 1**						**M 1' Vs M 1**	138
eGFR+	eGFR	0.97 (0.96-0.98)	<0.001	0.97 (0.96-0.98)	<0.001	<0.001	
proteinuria	Proteinuria	0.86 (0.75-0.99)	0.04	0.86 (0.73-1.02)	0.08		
**Model 1'**							125
eGFR+	eGFR	0.97 (0.96-0.98)	<0.001	0.97 (0.96-0.99)	<0.001		
proteinuria+	Proteinuria	0.96 (0.81-1.13)	0.59	1.13 (0.90-1.41)	0.3		
uL-selectin/Cr	uL-selectin/Cr	0.98 (0.96-0.99)	0.01	0.96 (0.94-0.99)	0.002		
**Model 2**						**M 2' Vs M 2**	162
SLEDAI+	SLEDAI	0.91 (0.83-0.99)	0.03	0.89 (0.81-0.99)	0.03	<0.001	
proteinuria	Proteinuria	0.98 (0.87-1.12)	0.8	1.01 (0.87-1.18)	0.85		
**Model 2'**							144
SLEDAI+	SLEDAI	0.96 (0.87-1.05)	0.36	0.95 (0.85-1.07)	0.42		
proteinuria+	Proteinuria	1.07 (0.93-1.23)	0.35	1.30 (1.07-1.58)	0.01		
uL-selectin/Cr	uL-selectin/Cr	0.98 (0.97-0.99)	0.01	0.96 (0.94-0.98)	<0.001		
**Model 3**						**M 3' Vs M 3**	131
eGFR+	eGFR	0.97 (0.96-0.98)	<0.001	0.97 (0.95-0.98)	<0.001	0.005	
SLEDAI+	SLEDAI	0.87 (0.78-0.97)	0.01	0.84 (0.74-0.96)	0.01		
proteinuria	Proteinuria	0.90 (0.78-1.04)	0.16	0.92 (0.78-1.10)	0.36		
**Model 3'**							124
eGFR+	eGFR	0.97 (0.96-0.98)	<0.001	0.97 (0.96-0.99)	<0.001		
SLEDAI+	SLEDAI	0.91 (0.81-1.03)	0.13	0.89 (0.77-1.03)	0.11		
proteinuria+	Proteinuria	0.96 (0.82-1.13)	0.64	1.14 (0.91-1.43)	0.27		
uL-selectin/Cr	uL-selectin/Cr	0.98 (0.97-1.00)	0.07	0.97 (0.95-0.99)	0.01		

OR odds ratio; **95% CI** 95% confidence interval; **LR test** likelihood ratio test; **eGFR** estimated glomerular filtration rate; **uL-selectin/Cr** urinary L-selectin levels adjusted by creatinine; **SLEDAI** Systemic Lupus Erythematosus Disease Activity Index;

[&]All models were adjusted for age, gender, and race. [#]LR tests were performed in two adjusted models; [*]AIC values were calculated in adjusted models, respectively.

Table S13. Comparison of remission and non-remission groups in the follow-up cohort of the Chinese cohort (n=20).

	CRR (n=9)	PRR (n=4)	NRR (n=7)	P-value[#]
Age at enrollment (years) (mean ± SD)	30.0±11.6	43.5±16.8	25.6±5.0	0.056
Female, n (%)	9 (100)	4 (100)	7 (100)	1.000
Follow-up duration (months, median (IQR))	14.2 (9.8)	14.2 (12.2)	12.9 (13.9)	0.913
**Renal pathology (ISN/RPS classification), n (%)**				
II	0 (0)	0 (0)	0 (0)	1.000
III	1 (11.1)	0 (0)	1 (14)	1.000
IV	5 (55.6)	0 (0)	2 (28.6)	0.165
V	1 (11.1)	1 (25)	1 (14)	1.000
III + V	2 (22.2)	1 (25)	1 (14)	1.000
IV + V	0 (0)	2 (50)	2 (28.6)	0.073
**Treatment during follow-up, n (%)**				
Prednisone	9 (100)	4 (100)	7 (100)	1.000
Hydroxychloroquine	7 (77.8)	3 (75.0)	3 (42.9)	0.409
Cyclophosphamide	4 (44.4)	2 (50)	3 (42.9)	1.000
Mycophenolate mofetil	4 (44.4)	2 (50)	2 (28.6)	0.72
Leflunomide	1 (11.1)	0 (0)	0 (0)	1.000
Azathioprine	3 (33.3)	0 (0)	0 (0)	0.266
Tacrolimus	0 (0)	0 (0)	1 (14)	0.55
Thalidomide	1 (11.1)	1 (25)	1 (14)	1.000
Tripterygium Glucosides	0 (0)	0 (0)	1 (14)	0.55
**Urine total protein/creatinine ratio (g/g) (median (IQR))**				
At the baseline	2.3 (2.3)	8.2 (10.2)	2.6 (4.5)	0.076
At the end point	0.1 (0.1)a	1.2 (0.6)b	2.6 (4.5)b	**0.001**
**Serum eGFR (mL/min/1.73m^2) (median (IQR))**				
At the baseline	129 (104)	78 (77)	142 (95)	0.368
At the end point	123 (50)	111 (55)	85 (106)	0.283
**Urine L-selectin levels (ng/mg) (median (IQR))**				
At the baseline	27.2 (44.2)	121 (120)	23 (15.4)	0.128
At the end point	3.4 (8.8)a	16.7 (52.9)a,b	55.0 (67.5)b	**0.028**

[#]P-value was obtained by Mann-Whitney U test for continuous variables and by Chi-Squared Test for numeration variables. Different letters (a or b) meant statistical differences after the Bonferroni correction. **CRR** complete renal remission; **PRR** partial renal remission; **NRR** no renal remission.

Table S14. Demographics and clinical characteristics of patients with chronic kidney diseases (n=33).

	**DN (n=13)**	**HN (n=3)**	**IgAN (n=5)**	**FSGS (n=3)**	**MN (n=5)**	**Others (n=4)**
**Ages (years)**	61.9 ± 10.9	65.7 ± 8.0	52.4 ± 14.0	53.0 ± 4.4	57.2 ± 13.3	59.8 ± 14.4
**Female, n (%)**	4 (31)	2 (67)	2 (40)	3 (100)	1 (20)	1 (25)
**CKD stages, n (%)**						
1	1 (7.7)	0 (0)	2 (40)	1 (33.3)	1 (20)	1 (25)
2	1 (7.7)	1 (33.3)	3 (60)	0 (0)	4 (80)	2 (50)
3	6 (46.2)	1 (33.3)	0 (0)	1 (33.3)	0 (0)	1 (25)
4	3 (23.1)	0 (0)	0 (0)	1 (33.3)	0 (0)	0 (0)
5	2 (15.4)	1 (33.3)	0 (0)	0 (0)	0 (0)	0 (0)
**Laboratory measurement, median (IQR)**						
**24h UP (g/24h)**	2.7 (3.9)	0.9 (NA)	1.6 (3.3)	2.9 (NA)	10.0 (6.7)	3.0 (5.5)
**Scr (umol/L)**	174 (111)	160 (NA)	98 (51)	104 (NA)	97 (32)	92 (86)
**eGFR-EPI (ml/min/1.73m^2)**	29 (20)	41 (NA)	86 (30)	54 (NA)	83 (26)	73 (50)
**Urine L-selectin/Cr (ng/mg), median (IQR)**						
	5.9 (11.7)	4.4 (NA)	9.1 (21.6)	8.6 (NA)	8.2 (12.6)	5.0 (12.0)

DN diabetic nephropathy; **HN** hypertensive nephropathy; **IgAN** IgA nephropathy; **FSGS** focal segmental glomerulosclerosis; **MN** membranous nephropathy; **CKD** chronic kidney disease; **24h UP** 24h urine protein; **Scr** serum creatinine; **eGFR** estimated glomerular filtration rate.

Table S15. Univariate logistic regression analyses for high AI (AI>6) in the Chinese cohort.

Variables	Odds Ratio (95%CI)	P-value
**Age, years**	1.00 (0.97-1,04)	0.86
**Gender**		0.43
Male	1 (reference)	
Female	0.48 (0.08-3.00)	
**SLE duration, years**	1.04 (0.97-1.12)	0.27
**LN duration, months**	1.01 (0.99-1.02)	0.45
**Low C3 and/or C4**		0.15
No	1 (reference)	
Yes	4.90 (0.57-42.63)	
**SLEDAI**	1.21 (1.08-1.36)	**0.001**
**rSLEDAI**	1.49 (1.20-1.86)	**<0.001**
**anti-dsDNA, IU/mL**	1,01 (0.99-1.02)	0.29
**24h urine protein, g/24h**	1.19 (0.04-1.36)	**0.01**
**eGFR, ml/min/1.73m^2**	0.98 (0.97-0.99)	**0.001**
**uL-selectin/Cr, ng/mg**	1.00 (1.00-1,01)	0.76

C3 complement 3; **C4** complement 4**; SLEDAI** Systemic Lupus Erythematosus Disease Activity Index; **rSLEDAI** renal SLEDAI; **anti-dsDNA** anti-dsDNA antibody; **eGFR** estimated glomerular filtration rate; **uL-selectin/Cr** urinary L-selectin levels adjusted by creatinine.

Table S16. Multivariate logistic regression analyses for high AI (AI>6) in the Chinese cohort.

**Parameter**	**Unadjusted**		**Adjusted**[&]	
	**OR (95% CI)**	**P-value**	**OR (95% CI)**	**P-value**
**M1**				
Proteinuria	1.23 (1.05-1.43)	**0.01**	1.28 (1.06-1.53)	**0.01**
uL-selectin/Cr	1.00 (0.99-1.00)	0.37	1.00 (0.99-1.00)	0.35
**M2**				
SLEDAI	1.25 (1.10-1.43)	**<0.001**	1.36 (1.15-1.60)	**<0.001**
uL-selectin/Cr	1.00 (0.99-1.00)	0.22	1.00 (0.99-1.00)	0.24
**M3**				
rSLEDAI	1.54 (1.21-1.95)	**<0.001**	1.61 (1.24-2.09)	**<0.001**
uL-selectin/Cr	1.00 (0.99-1.00)	0.39	1.00 (0.99-1.00)	0.51
**M4**				
eGFR	0.98 (0.97-0.99)	**0.001**	0.98 (0.96-0.99)	**<0.001**
uL-selectin/Cr	1.00 (1.00-1.01)	0.45	1.00 (1.00-1.00)	0.48

OR odds ratio; **95% CI** 95% confidence interval; **LR test** likelihood ratio test; **eGFR** estimated glomerular filtration rate; **uL-selectin/Cr** urinary L-selectin levels adjusted by creatinine; **SLEDAI** Systemic Lupus Erythematosus Disease Activity Index; [&]All models were adjusted for age, gender, SLE disease duration and LN disease duration.

Table S17. Multivariate logistic regression analyses for high AI (AI>6) in the two cohorts (n=119).

**Parameter**	**Unadjusted**		**Adjusted[&]**	
	**OR (95% CI)**	**P-value**	**OR (95% CI)**	**P-value**
**M1**				
Proteinuria	0.79 (0.67-0.93)	**0.005**	0.69 (0.56-0.85)	**<0.001**
uL-selectin/Cr	1.00 (0.99-1.01)	0.97	1.00 (1.00-1.01)	0.31
**M2**				
SLEDAI	0.85 (0.77-0.94)	**0.001**	0.84 (0.76-0.94)	**0.001**
uL-selectin/Cr	1.00 (0.99-1.01)	0.86	1.00 (0.99-1.01)	0.91
**M3**				
rSLEDAI	0.83 (0.74-0.93)	**<0.001**	0.78 (0.68-0.90)	**<0.001**
uL-selectin/Cr	1.00 (0.99-1.01)	0.64	1.00 (0.99-1.01)	0.67
**M4**				
eGFR	1.01 (1.00-1.02)	0.07	1.01 (1.00-1.02)	**0.04**
uL-selectin/Cr	0.99 (0.99-1.00)	0.12	1.00 (0.99-1.00)	0.19

OR odds ratio; **95% CI** 95% confidence interval; **LR test** likelihood ratio test; **eGFR** estimated glomerular filtration rate; **uL-selectin/Cr** urinary L-selectin levels adjusted by creatinine; **SLEDAI** Systemic Lupus Erythematosus Disease Activity Index; [&]All models were adjusted for age, gender, and race.

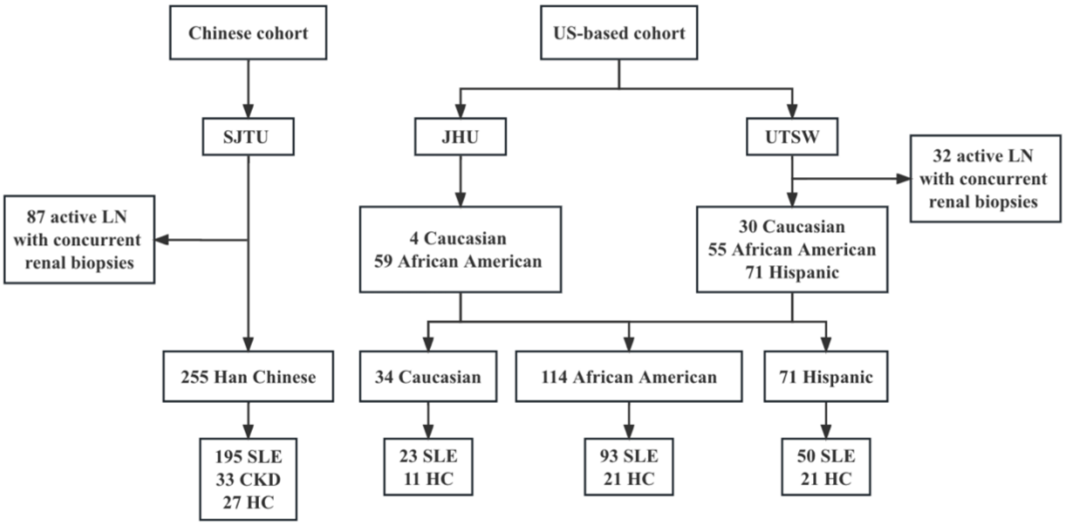

Figure S1. Flow diagram of the study. The two groups of active LN patients with concurrent renal biopsies were included in the 255 Han Chinese cohort and 156 UTSW cohort, respectively.

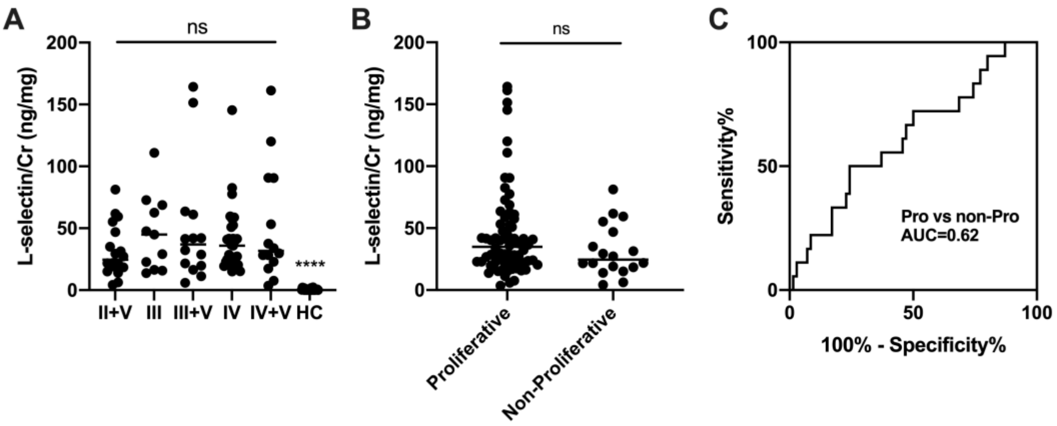

Figure S2. uL-selectin levels in LN patients with concurrent renal biopsies parsed by pathology class in the Chinese SLE patients (n=87) and healthy controls (n=27). **(A)** uL-selectin levels were elevated in each LN class compared to healthy controls. Kruskal–Wallis test with Dunn post hoc tests. **(B)** uL-selectin levels were comparable between patients with proliferative LN (III±V & IV±V) and non-proliferative LN (II & V). Mann-Whitney U test. **(C)** ROC curve analysis for uL-selectin levels to discriminate proliferative LN from non-proliferative LN (p=0.13). ****P<0.0001

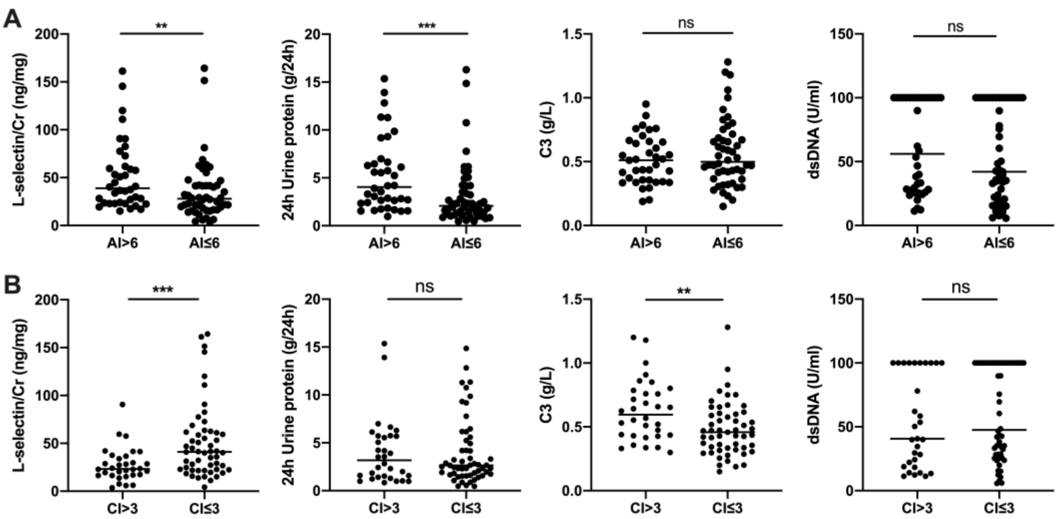

Figure S3. Subgroup analyses for AI **(A)** and CI **(B)** in the Chinese cohort. **(A)** uL-selectin levels and 24h urine protein could discriminate high AI (AI>6) and low AI (AI≤6). **(B)** uL-selectin levels and C3 levels could discriminate high CI (CI>3) and low CI (CI≤3). **AI** activity index; **CI** chronicity index. Mann-Whitney U test; **P*<0.05, ***P*<0.01, ****P*<0.001.

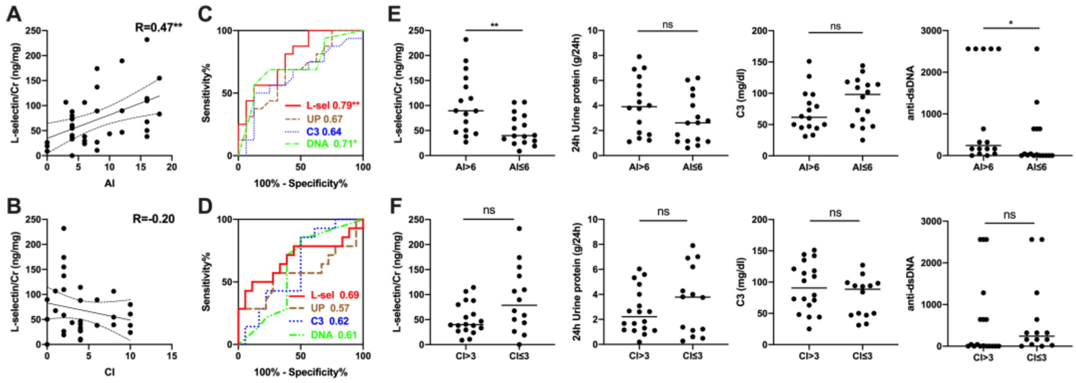

Figure S4. Correlation analyses **(A, B)**, ROC curve analyses **(C, D)** and subgroup analyses **(E-F)** between uL-selectin and renal disease indexes in the US-based cohort. **(A, B)** uL-selectin levels positively correlated with AI and negatively correlated with CI (not attaining significance). **(C, D)** uL-selectin levels could discriminate high AI (AI>6) and low AI (AI≤6) but could not discriminate high CI (CI>3) and low CI (CI≤3). **AI** activity index; **CI** chronicity index; **L-sel** urinary L-selectin/Cr levels; **UP** 24h proteinuria; **R** Spearman's correlation coefficient. Mann-Whitney U test, **P* < 0.05, ***P* < 0.01.

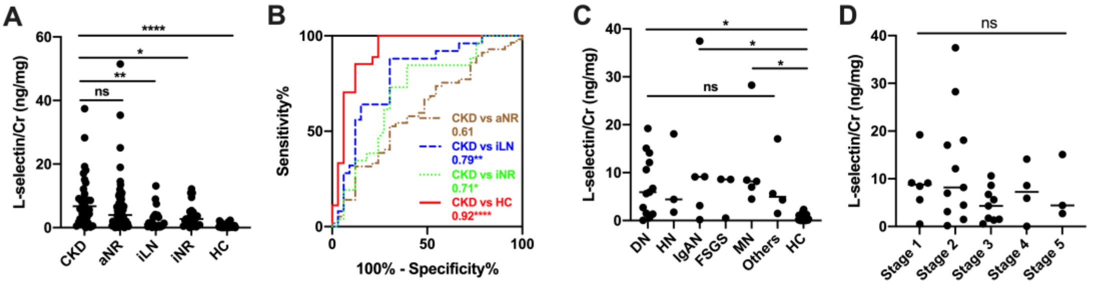

Figure S5. (A, B) Comparison of uL-selectin levels in CKD subgroup with aNR patients, iLN patients, iNR patients and HC. uL-selectin levels in patients with variable types of CKD compared with HC **(C)** as well as among different CKD stages **(D)**. Kruskal-Wallis test with Dunn post hoc tests; $*P < 0.05$, $**P < 0.01$, $****P < 0.0001$.
